# Single-Dose of Testosterone and the *MAOA* VNTR Polymorphism Influence Emotional and Behavioral Responses in Men During a Non-social Frustration Task

**DOI:** 10.3389/fnbeh.2020.00093

**Published:** 2020-06-25

**Authors:** Lisa Wagels, Mikhail Votinov, Philippa Hüpen, Sonja Jung, Christian Montag, Ute Habel

**Affiliations:** ^1^Department of Psychiatry, Psychotherapy and Psychosomatics, Medical Faculty, Uniklinik, RWTH Aachen, Aachen, Germany; ^2^Institute of Neuroscience and Medicine 10, Research Center Juelich, Juelich, Germany; ^3^Department of Molecular Psychology, Institute of Psychology and Education, Ulm University, Ulm, Germany

**Keywords:** hormone application, monoamine oxidase A polymorphism, anger, testosterone, provocation

## Abstract

Previous studies suggest that testosterone and several neurotransmitters might interactively influence human aggression. The current study aimed to test potential interactions of a genetic variation linked to the catabolism of serotonin, dopamine, and norepinephrine and exogenous testosterone on the reaction towards non-social provocation. In total, 146 male participants were genotyped for a prominent polymorphism of the monoamine oxidase A (*MAOA*) gene resulting in a short and long variant. Participants completed a non-social frustration task after receiving either testosterone or a placebo gel in a double-blind set-up. Participants performed a non-social frustration task, where they had to direct a virtually moving ball into a barrel by pulling a joystick (neutral block). During a frustration block, the joystick repeatedly did not respond to participants’ reactions thereby causing failed trials to which participants reacted with increased anger and stronger pulling of the joystick. We analyzed the effect of testosterone administration on emotion and behavior in individuals who either carried a low (L) or high (H) activity *MAOA* variant. Testosterone administration increased provocation-related self-reported anger and abolished the association between trait aggression and joystick deflection in the frustration block. In *MAOA*-H carriers endogenous testosterone levels at baseline were associated with increased joystick deflection in both blocks. There was, however, no interaction of testosterone administration and genotype. Although preliminary, the results rather indicate independent influences of exogenous testosterone administration and *MAOA*, but support an interaction of endogenous testosterone levels and *MAOA* genetics in a frustration task. The administration of testosterone seems to act on the subjective emotional experience in a provoking situation, while endogenous testosterone levels increased pulling impulses only in carriers of the *MAOA*-H variant.

## Introduction

The neurocognitive system “*frustrative nonreward*” is one of the subdomains defined by the research domain criteria (RDoC) that contribute to aggression. Externalizing behaviors, for example, seem to be characterized by deficits in processing the omission of a predicted reward (Gatzke-Kopp et al., 2009). As one of five subdomains of the negative valence system, the concept of frustrative non-reward describes the situation in which an individual is impeded from obtaining a previously available award. This becomes especially relevant if frustration appears after repeated or sustained effort, which stays unrequited. Typical effective response within such a frustrating situation is anger (Novaco, 2016), which ultimately can lead to aggression (Berkowitz, 1989). Other important concepts in the negative valence system, which may activate a defensive aggressive response, are an acute or sustained threat, and—more ambiguous, distant, or uncertain—potential harm (Kozak and Cuthbert, 2016). Each of these conceptual domains may be defined by specific neurobiological substrates including genetic or hormonal modulators and reflect the full range of human behavior from normal to abnormal. The current study aims to characterize the influence of a genetic polymorphism and the steroid hormone testosterone and their potential interaction on affective and behavioral responses to sustained frustration in healthy young males.

While aggression is typically associated with social threat or provocation, frustration can appear in social and non-social contexts. The RDoC framework declares the point subtraction aggression paradigm (PSAP) as an experimental task to assess aggression in the framework of frustrative non-reward (Del Pozzo et al., 2019). This task has a clear social component, as an ostensible opponent subtracts points or money from the participant who thereby loses an already achieved reward. Other paradigms that were more recently developed, however, do not present a social component (e.g., Panagiotidis et al., 2017; Tseng et al., 2017; Angus and Harmon-Jones, 2019; Seymour et al., 2020). In these tasks, participants try to achieve a reward, but either receive rigged feedback about their performance and the consequential loss of the reward or are impeded to fulfill the task due to technical manipulations. Across all paradigms, these manipulations lead to increased anger or irritability (Seymour et al., 2020).

Testosterone, similarly to findings in other species, is assumed to enhance human aggression especially in the presence of a social challenge (Eisenegger et al., 2011; Wingfield, 2016). However, research findings are heterogeneous and do not always support the enhancement of aggression *via* testosterone. Depending on the context and individual characteristics, testosterone seems to promote either prosocial or antisocial behaviors (Zilioli and Bird, 2017; Carré and Archer, 2018). To name just a few research findings, testosterone affected punishment behavior depending on the preceding fairness of an ostensible opponent (Dreher et al., 2016; Wagels et al., 2018); it increased reciprocity when participants were trusted with high investments in a trust game (Boksem et al., 2013) and cooperation with in-group members during the intergroup competition (Reimers and Diekhof, 2015); and finally, it promotes utilitarian choices (Carney and Mason, 2010; Arnocky et al., 2017) or behaviors that ensure a high social status (Eisenegger et al., 2010; Dreher et al., 2016). In a previously reported experiment on testosterone administration in healthy men, our group could show that males in the testosterone group compared to the placebo group adapted their behavior strategically to that of the opponent: they selected higher punishments if their ostensible opponent stole high amounts of money from their account, whereas they responded less aggressively when provocation was low or zero (Wagels et al., 2018). In a similar social provocation experiment, testosterone administration increased aggressive responses to provocation especially in individuals with low regulatory abilities, such as high impulsive men, but did not affect men scoring low on dominant or impulsive traits (Carré et al., 2017). Thus, context and personality seem to moderate the effects of testosterone administration.

However, despite the extensive research in the field of social endocrinology and the association of testosterone with a threat, provocation and aggression, a surprisingly small number of studies investigated its effects in a frustration context. Findings in our group suggest that testosterone administration increases angry responses to sustained frustration, which was induced through a technical manipulation prompting the participant to repeatedly fail the task (Panagiotidis et al., 2017). In this task, participants pulled a joystick to direct a moving ball into a virtual bottle, but a manipulation disrupted the joystick function. Thus, participants lost the trial and the associated monetary reward. While participants were found to pull the joystick stronger in the frustration block, testosterone administration did not increase this behavioral response. Interestingly data of Tseng et al. (2019), measuring neural responses in a frustration task, suggest that different neurocognitive circuits underlie the direct response to frustrating feedback and the behavioral reaction afterward. They conclude that especially the latter response is affected by re-orientation and top-down control. Hence, testosterone administration may primarily affect the emotional rather than the behavioral response following continuous frustration.

The X-chromosome-linked *MAOA* gene codes for the monoamine oxidase A, an enzyme that degrades serotonin, norepinephrine, and dopamine. The promoter region of the gene is characterized by a variable number of 30 base pair tandem repeats, usually comprising 2-, 3-, 3a-, 4-, 5-repeat alleles (Sabol et al., 1998; Deckert et al., 1999) which affect the expression of the gene. While the role of the 5-repeat allele is not yet entirely understood (Kim-Cohen et al., 2006), the shorter alleles (2 or 3 repeats) are associated with reduced transcriptional efficiency compared to the long alleles (3a or 4 alleles; Sabol et al., 1998; Deckert et al., 1999). Several studies show that this transcriptional efficiency influences proxies of aggression and anti-social behavior (Buckholtz and Meyer-Lindenberg, 2008; Godar et al., 2016). Instead of referring to the allele length, we will thus refer to the transcription characteristics using the abbreviation *MAOA*-L for the low transcriptional variants and *MAOA*-H for the high transcriptional variants. This classification is commonly used in the scientific community. Interestingly, empirical evidence points to a sexually-dimorphic role of the *MAOA* VNTR (Reif et al., 2012; Perry et al., 2017). In particular, males, as opposed to females, have an increased tendency to show impulsive aggression if they carry a variant linked to lower *MAOA* activity (Godar et al., 2016). While not supported in all studies (Schlüter et al., 2016), in males these low activity variants are mostly related to increased aggression after provocation (McDermott et al., 2009; Kuepper et al., 2013) or social exclusion (Gallardo-Pujol et al., 2013). Findings on another *MAOA* variant (single nucleotide polymorphism, rs1465108) suggest that reduced control abilities could be an underlying mechanism since researchers found increased impulses to negative affect in these individuals (Chester et al., 2015). Neuroimaging studies suggest that the *MAOA* polymorphism might affect anger control (Denson et al., 2014) and anger reactivity (Alia-Klein et al., 2009).

The different effects of the *MAOA* VNTR observed in males and females may be influenced by sex-specific hormones such as testosterone. The concentration of the steroid hormone testosterone is much higher in males. Consequently, it could be assumed that high testosterone levels are the basis for the aggression enhancing effect observed in *MAOA*-L carriers. A previous study indicated that in individuals carrying a genetic variant associated with lower enzyme activity, higher levels of CSF testosterone levels were associated with increased Brown–Goodwin scores measuring lifetime aggression levels (Sjöberg et al., 2008). This may suggest an interaction of both factors motivating the current research question. To test, if such an interaction can explain sex differences, a mixed sample of males and females would be required. However, since the administration of Testim (testosterone gel) in females is currently not allowed in Germany and may have distinct effects in males and females *per se*, the current study focused on males only. Besides the notification of sex differences, other findings motivate to investigate a potential interaction of testosterone and *MAOA* activity. Testosterone and serotonin, which is degraded by *MAOA*, seem to have a close relationship (Perfalk et al., 2017). *MAOA* expression might influence aggression in interaction with testosterone more indirectly *via* serotonin (Birger et al., 2003; Montoya et al., 2012). Recently, it has also been shown that the effects of exogenous testosterone are moderated by variations in the dopaminergic system (Losecaat Vermeer et al., 2020). As suggested by the authors, testosterone might act *via* androgen-dependent actions on striatal dopamine to influence status-seeking motivation. Similar mechanisms might modulate reactions that emerge when an individual is impeded from obtaining a previously available reward. Finally, we observed that individuals who received a single dose of testosterone showed increased risk-taking if they were carriers of a *MAOA* variant associated with low activity (Wagels et al., 2017b). In a social provocation task, those individuals showed higher anger paralleled by reduced brain activity in the cuneus during a social provocation task if they did not receive testosterone (Wagels et al., 2019b). Here testosterone administration increased aggressive behavior independent of the *MAOA* variant in response to high provocation compared to low provocation (Wagels et al., 2018). Thus, instead of a biological interaction, that depicts a risk factor for aggression, *MAOA* and testosterone may have similar effects as distinct entities that nevertheless partially overlap and activate similar brain regions. If both factors have similar effects but do not directly interact, we might expect different patterns depending on the measured variable or the context: For instance, testosterone might affect the emotional reactivity to provocation, while the *MAOA* VNTR influences behavior in response to provocation.

In the current study, we aim to test for a possible interaction of testosterone administration with the *MAOA* VNTR during a non-social frustration task in which we previously found that testosterone increased the affective response anger. Concerning previous findings on the task (Panagiotidis et al., 2017; Wagels et al., 2019b), we assume an increase of anger and increased joystick amplitudes during the frustration block across participants. We reanalyzed the data since we specifically wanted to test if the *MAOA* VNTR interacts with the exogenously modulated or endogenous testosterone levels in a non-social frustration context, which has not been tested before. Merging two parallel data sets, we analyzed a larger sample than used in the previous study (Panagiotidis et al., 2017). Concerning the administration of testosterone and the influence of the *MAOA* VNTR, we suggest two opposing hypotheses (1 and 2) concerning the exogenous testosterone administration.

(1) *Interaction effect hypothesis*: in case of biological interactions that are influenced by exogenous testosterone manipulation, we assume an effect of testosterone administration on anger and increased behavioral impulses during the frustration task in carriers of the low activity *MAOA* variant (*MAOA*-L).
(2) *Separate mechanisms hypothesis*: testosterone and *MAOA* independently influence anger and behavior in the frustration task. We assume that testosterone administration will increase anger and that *MAOA-*L carriers will show increased behavioral reactions in the frustration task. We do not expect an interaction of testosterone administration and the MAOA variant.
(3) Additionally, we test, if endogenous testosterone levels (baseline levels before administration) interact with the MAOA variant, influencing anger and behavior in the frustration task.
(4) Since personality traits have been shown to influence the effects of testosterone administration on behavior, we test the influence of trait aggression, assuming that increased trait aggression enhanced anger and frustration related behavioral impulses more after testosterone administration.

## Materials and Methods

### Sample

Participants were recruited by postings, advertisements in lectures, and online platforms of the university. General inclusion criteria were age above 18, fluent German language, and being male. In total, 146 male participants (origin: 95% Caucasian, 5% other) gave oral and written informed consent to take part in the study. Exclusion criteria for study participation included current or previous psychiatric diagnosis assessed by the structured clinical interview (SCID I) for DSM IV (Wittchen et al., 1997). Further exclusion criteria were neurological problems, contraindications against magnetic resonance imaging (MRI; additional functional MRI measures were performed following the here reported experiment), left-handedness, high blood pressure, current nicotine consumption, and known allergic reactions to the testosterone gel. Participants were asked to refrain from alcohol consumption 24 h before participating in our study. One participant was excluded due to additional MR contraindications. One outlier had to be excluded because this participant did not perform the task adequately (no response in more than half of the trials). The results are therefore presented for 144 male participants. Of these 144 participants, 75 received testosterone (T), and 69 received placebo (PL). Both groups (T, PL) did not differ in age (*M*_T_ = 24.34, *M*_PL_ = 24.12; *t*_(142)_ = −0.35, *p* = 0.727). Regarding the *MAOA* VNTR polymorphism participants were grouped as *MAOA*-L (low activity variants) and *MAOA*-H (high activity variants).

### Ethics Approval

Participants gave oral and written informed consent to participate in the study. All study procedures were compliant with the latest version of the Code of Ethics of the World Medical Association (Declaration of Helsinki). Additionally, the Medical Ethics Committee of the Medical Faculty, RWTH Aachen University approved of the described study procedures.

### Procedure

Data analyses presented here are based on two merged data sets in which participants received transdermal testosterone application before performing the behavioral task reported here. Both studies included two parts, which were separated by a 1.5 h break. The first part of both studies was identical, while after the break, participants of one study additionally received arginine vasopressin before performing several tasks in the MRI scanner. In the other study, participants performed the same tasks in the MRI scanner but did not receive arginine vasopressin before the measurement. Since this administration of arginine vasopressin was not related to the reported data, which were assessed in the first part of the experiment, both data sets are merged for the current analyses.

In both studies, participants arrived in the early afternoon because of a reduced individual variability of testosterone levels at this time (Dabbs et al., 1990). A first blood sample was collected to measure hormonal baseline levels. Subsequently, either a placebo gel (hydrogel conventionally used for ultrasound) or a testosterone gel [5 g Testim™, containing 50 mg of the active agent (17-β hydroxyandrost-4-en-3-one)] was applied on the skin of the upper back and shoulders of the participant. Due to a neutral packaging experimenter and participants were blinded to group allocation (double-blind setup). Approximately half of the participants then provided a buccal swap for genotyping; all other participants gave a buccal swap at a prior screening. DNA from buccal mucosa cell samples was analyzed in a collaborate laboratory (to analyze the *MAOA* VNTR and polymorphic elements of the serotonin transporter gene (SLC6A4; Molecular Psychology, Ulm, Germany; for further descriptions see Wagels et al., 2017b and Supplementary Material). Variation in the SLC6A4 was not further considered for the analysis as this would have resulted in small groups with unequal group sizes.

The experimental task started after approximately 200–220 min post testosterone/placebo application. This time delay was chosen because the first peak of serum testosterone increase was detected here in a study that tested the effects of Testim™ in hypogonadal males (Marbury et al., 2003). Previously, we could ensure a significant increase in plasma testosterone levels of the T compared to the PL group after 3 and a half hours up to approximately 6 h (Wagels et al., 2017a,b). After the experimental task reported here, a second blood sample was taken to measure hormonal levels (T1). Subsequently, participants in both studies underwent an MRI scan while performing a modified Taylor Aggression Paradigm and a scanner compatible version of the Balloon Analogue Risk Task.

Additionally, participants completed several questionnaires related to personality traits. In both studies, trait aggression was assessed with the Buss Perry Aggression Questionnaire (BPAQ; Buss and Perry, 1992).

### Technical Provocation Task

In the Technical Provocation Paradigm (TPP; Panagiotidis et al., 2017; Wagels et al., 2019a), individuals are instructed to direct a horizontally moving ball into a barrel by pulling a joystick to win virtual gold coins. Participants are informed that each virtual coin will equal 20 real Euro Cents. The paradigm consists of two blocks each lasting 7 min including 40 trials. Unknown to the participant, the joystick does not respond to the participants’ actions in 12 trials (frustration block). Since the moving ball does not drop down (vertically), the ball cannot be successfully placed in the barrel and a potential reward is missed. Also, a provoking message is given (“Please move the joystick!”) following these manipulated trials before the next trial starts. Participants rate their emotions [emotional state rating, ESR (Schneider et al., 1994)] after each block. In this standardized emotion measure participants rate their happiness (How happy do you feel right now?), their anger (How angry do you feel right now?), their sadness (How sad do you feel right now?), their surprise (How surprised do you feel right now?) and their anxiety (How anxious do you feel right now?) on a five-point Likert-like scale from 1 “not at all” to 5 “extremely”. Also, the joystick movement, measuring the pulling (maximal pulling equaled 200 mm) of the participant, is assessed during the complete experiment.

### Statistical Procedures

Regarding the distribution of genetic variants and the treatment allocations, Chi-square tests of homogeneity were conducted. Hormonal levels were log-transformed (due to a skewed distribution) and subsequently analyzed using a repeated-measures ANOVA including time (t0, before gel application; T1, after the task) as within-subject factors and treatment (T, PL) and genotype (*MAOA*-L, *MAOA*-H) as between-subject factors. For validation of the testosterone manipulation, plasma testosterone levels should differ between T and PL at T1 (but not at baseline).

Further analyses were performed in R^1^. We calculated two general linear mixed models using the package lme4 including a random intercept (ID) and the following fixed factors: group (PL, T), genotype (*MAOA*-L, *MAOA*-H), condition (neutral, frustration), log-transformed baseline testosterone (t0) and trait aggression (BPAQ). In addition to the main effects, we specified interactions (condition*t0, genotype*t0, condition*group, condition*genotype, BPAQ*group, BPAQ*genotype, condition*BPAQ, genotype*group, genotype*group*BPAQ). In the first model, the dependent variable was subjective anger as assessed *via* the ESR during the paradigm presentation at the end of each block. In the second model, the dependent variable was the peak amplitude (maximal deflection) of the joystick within a trial which was calculated as an absolute value and averaged across each block.

## Results

There was no significant difference in the distribution of *MAOA*-L and *MAOA-H* carriers concerning the treatment allocation (Table 1), (1) = 0.31, *p* = 0.738.

**Table 1:** Number of participants per group and genotype.

	PL	T
MAOA-L	27	31
MAOA-H	42	44

### Hormonal Levels

**Figure 1:**
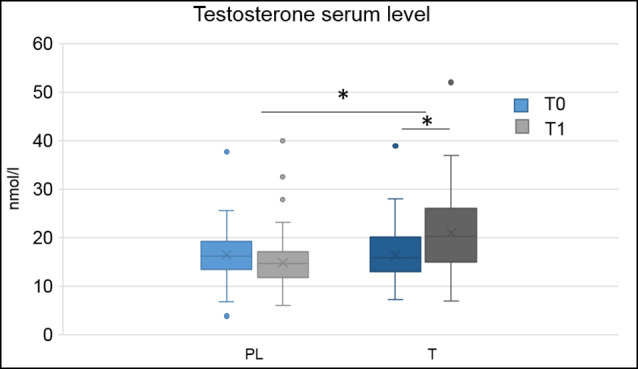
Boxplots, depicting minimum, maximum, median, first quartile and third quartile of hormonal serum levels at baseline (t0) and directly after the applied paradigm (T1) for the placebo (PL) and testosterone (T) group, *p < 0.05.

Blood serum testosterone levels were higher in the T group compared to the PL group, *F*_(1,133)_ = 10.78, *p* = 0.001, = 0.08 and at time point T1 compared to time point t0, *F*_(1,133)_ = 6.46, *p* = 0.012, = 0.05. There was an interaction of hormonal treatment and time, *F*_(2,132)_ = 56.63, *p* < 0.001, = 0.30. *Post hoc* comparisons demonstrated that treatment groups did not significantly differ from each other at t0 (*p* = 0.730), but at T1, *F*_(1,132)_ = 33.71, *p* < 0.001, = 0.20, with higher testosterone blood serum levels in the T group (see Figure 1). There were no main effects or interactions of the genetic variant (all *p* > 0.05).

### Task

**Figure 2:**
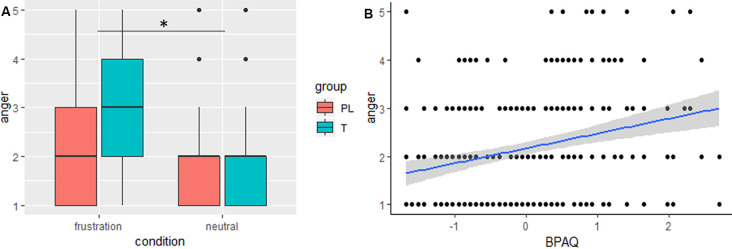
**(A)** Boxplots, depicting minimum, maximum, median, first quartile, and the third quartile of subjective anger ratings after the frustration and after the neutral block are indicated separately for the placebo (PL) and testosterone (T) group. **(B)** The slope (blue) and confidence interval (gray) of trait aggression (BPAQ) is depicted predicting subjective anger, *p < 0.05.

The general linear mixed model on anger showed significant effects for condition, trait aggression (BPAQ), and the interaction of condition by group (see Table 2 for an overview on the fixed effects on the anger model). Anger was increased in the frustration block compared to the neutral block. *Post hoc* tests on the group by condition interaction showed an increase of anger after the frustration block both in the PL and in the T group. Groups did not differ in the neutral or frustration block, but the increase of anger was higher in the T group (see Figure 2A). Trait aggression was positively related to subjective anger (see Figure 2B).

**Table 2:** Effects on subjective anger during the frustration task.

	*df* (num)	*df* (den)	*F*	*p*	lower CI	upper CI
Condition	1	139	39.03**	<0.001	0.23	0.44
Group	1	134	0.72	0.347	−0.20	0.08
BPAQ	1	134	14.93**	<0.001	0.15	0.43
Genotype	1	134	0.02	0.900	−0.13	0.15
t0	1	134	0.38	0.539	−0.19	0.10
condition*t0	1	139	1.28	0.247	−0.16	0.04
t0*genotype	1	134	<0.001	0.987	−0.14	0.14
Condition*group	1	139	4.02*	0.047	−0.21	−0.003
Condition*genotype	1	139	0.49	0.485	−0.07	0.14
Group*genotype	1	134	1.15	0.234	−0.11	0.17
Group*BPAQ	1	134	0.22	0.640	−0.06	0.22
Genotype*BPAQ	1	134	1.28	0.260	−0.08	0.12
Condition*BPAQ	1	139	0.15	0.700	−0.23	0.05
Group*genotype*BPAQ	1	134	0.04	0.840	−0.16	0.13

t0, log-transformed baseline testosterone levels; BPAQ, trait aggression score. *p < 0.05; **p < 0.001.

**Figure 3:**
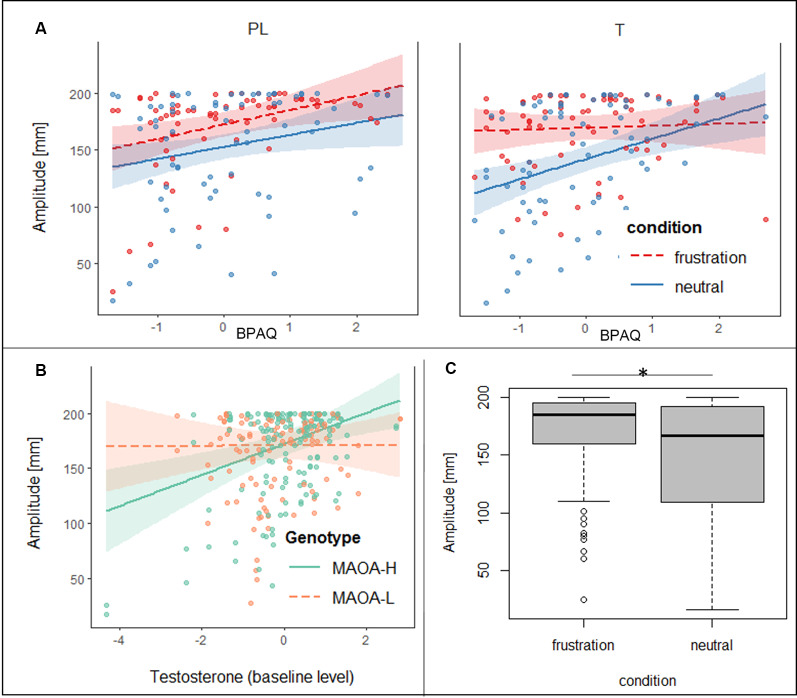
**(A)** Slopes and confidence intervals of trait aggression (BPAQ) are indicated separately for the placebo (PL) and testosterone (T) group in the frustration block (red) and the neutral block (blue) predicting peak amplitudes of the joystick movement. **(B)** Slopes and confidence intervals of baseline testosterone levels (log-transformed, in t0) are depicted for *MAOA*-H (green) and *MAOA-L* (orange) predicting peak amplitudes of the joystick movement. **(C)** Boxplots, depicting minimum, maximum, median, first quartile, and the third quartile of the averaged peak amplitudes of the joystick movement is depicted for the neutral and frustration block, *p < 0.05.

Regarding the joystick amplitude, the general linear mixed model showed significant effects for condition, trait aggression, baseline testosterone (t0), the interaction of BPAQ and condition, the interaction of genotype and t0 and the three-way-interaction of the group, condition, and BPAQ (see Table 3 for an overview on the fixed effects of the joystick amplitude model). The amplitude was higher in the frustration block compared to the neutral block (see Figure 3C). The BPAQ and t0 were positively related to the joystick amplitude. To further disentangle the interaction of the BPAQ and condition, the slope of the BPAQ was tested separately for each condition, revealing a significant association in the neutral condition (Estimate = 13.21, SE = 3.52, *t* = 3.76, *p* < 0.001) but not in the frustration condition (Estimate = 6.27, SE = 3.52, *t* = 1.78, *p* = 0.080). However, this was further influenced by the treatment group: In the PL group, there was a significant positive relationship in the neutral (Estimate = 12.28, SE = 4.82, *t* = 2.55, *p* = 0.01) and frustration condition (Estimate = 10.23, SE = 4.82, *t* = 2.12, *p* = 0.03), while in the T group there was only a significant positive relationship in the neutral block (Estimate = 15.95, SE = 5.07, *t* = 3.15, *p* < 0.001), but no significant relationship in the frustration block (Estimate = 0.73, SE = 5.07, *t* = 0.14, *p* = 0.89; see Figure 3A).

**Table 3:** Effects on the joystick deflection (peaks) during the frustration task.

	*df* (num)	*df* (den)	*F*	*p*	lower CI	upper CI
Condition	1	139	67.61**	<0.001	9.40	15.19
Group	1	134	1.08	0.301	−2.74	9.31
Genotype	1	134	0.07	0.785	−5.19	6.92
BPAQ	1	134	8.29*	0.005	3.16	15.51
t0	1	134	5.28*	0.023	1.25	13.36
Condition*group	1	139	1.51	0.221	−4.64	1.04
Condition*genotype	1	139	3.21	0.075	−5.61	0.22
Condition*BPAQ	1	139	5.01*	0.027	−6.15	−0.44
Group*BPAQ	1	134	0.21	0.645	−4.56	7.48
Genotype*BPAQ	1	134	0.56	0.456	−3.70	8.47
Genotype*t0	1	134	6.52^*	0.012	2.16	14.80
Group*t0	1	134	2.27	0.134	−11.38	1.32
Group*genotype	1	134	0.08	0.773	−6.98	5.14
Group*genotype*BPAQ	1	139	8.58*	0.004	1.46	7.18

t0, log-transformed baseline testosterone levels; BPAQ, trait aggression scores, *p < 0.05; **p < 0.001.

The interaction of genotype by baseline testosterone showed that testosterone was not related to the joystick amplitude in *MAOA*-L carriers (Estimate = −0.96, SE = 4.79, *t* = −0.20, *p* = 0.84), but it was positively related to the joystick in *MAOA-H* carriers (Estimate = 16.00, SE = 4.41, *t* = 3.62, *p* < 0.001; see Figure 3B).

## Discussion

The current study aimed to investigate whether testosterone administration and the *MAOA* VNTR have interactive effects on emotion and behavior during a frustration task. Alternatively, they might exert separate effects on emotion and behavior in the frustration task. Indeed, the testosterone administration effect on anger reported previously (Panagiotidis et al., 2017) was present in the analysis of this merged and larger data set, and this was not modulated by the *MAOA* VNTR. In contrast, the *MAOA* VNTR modulated the relationship of endogenous testosterone levels and behavior in the task, namely the joystick deflection. Individuals with high baseline T levels and the high active (*MAOA*-H) variant pulled the joystick stronger, than those with lower baseline testosterone levels. While our findings support previous studies that demonstrated genetic and hormonal effects in the context of anger provocation, the current results do not support that exogenous testosterone administration affects anger depending on the *MAOA* VNTR variant. While the administration of testosterone seems to influence the emotional reaction to frustration, anger, the *MAOA* VNTR seems to moderate the effect endogenous baseline testosterone levels have on behavior in the frustration task. Moreover, this behavioral response is altered not as a specific response to experimentally manipulated frustration but the effect appears across the task. This may point to the *hypothesis of separate mechanisms* regarding exogenous testosterone and the *MAOA* VNTR, but a potential interactive mechanism of endogenous testosterone and the *MAOA* VNTR.

On the one hand, the current findings underline the modulatory role of testosterone on anger outside of a social context as already shown in a previous analysis of a subsample (study 1, using testosterone application only; Panagiotidis et al., 2017). The findings in this merged and much larger data set substantiate the influence of testosterone during sustained frustration in a non-social context on anger development. The non-social element is particularly interesting since the effects of testosterone are frequently described in contexts such as social provocation or competition (Carré and Archer, 2018). That is likely because two popular hypotheses are usually applied to predict the effect of testosterone, both grounded in the social context: First, the challenge hypothesis (Wingfield et al., 1990) states that testosterone levels rise towards a social challenge thereby promoting aggressive responses towards a social challenger. Second, the status hypothesis refers to face-to-face groups arguing that high levels of endogenous testosterone may encourage behavior intended to dominate—to enhance one’s status over other people (Mazur and Booth, 1998). These hypotheses can explain various behavioral effects including aggressive as well as prosocial behavior (Boksem et al., 2013; Dreher et al., 2016) but they cannot be allied to non-social contexts and they rather neglect the influence of testosterone on the emotional system *per se*.

The current findings indicate that testosterone affects the emotional response to provocation independent of a social opponent that may challenge their promised reward, aim, status, or territory. Certainly, it is possible and even probable that participants felt challenged, when they did not receive a reward or when their joystick did not work. The behavioral differences, measured by the deflection of the joystick amplitude, may thus reflect the reaction to an acute challenge. However, the emotional rating was assessed after the completed block and thereby very likely represents the effect of the sustained frustration, not the acute challenge. Nevertheless, a competitive context was present, which might catalyze the testosterone effect as well. Our results furthermore show that this effect on the emotional component may not be limited to anger, since we also observe a reduction in happiness, with a stronger reduction in the group that received testosterone (see Supplementary Material).

Both, the competition induced challenge, and the acute loss of reward, which over time results in sustained frustration may require frustration tolerance or emotion regulation, which, if reduced, may lead to increased anger or conversely inhibit a reduction (Hawkins et al., 2013). Findings of an early study on circulating testosterone in adolescents suggest that high testosterone may actually lower frustration tolerance (Olweus et al., 1988). Emotion regulation might have been affected by testosterone administration in the current study as well. However, the absence of behavioral effects on the joystick pulling suggests that, even in the light of higher anger and reduced positive effect, testosterone administration did not increase aggressive behavioral tendencies, i.e., the joystick pulling. This might be an indication of high behavioral control despite increased frustration related to anger. Alternatively, the absence of a direct opponent or target for an aggressive retaliation may contribute to the missing behavioral effect. In terms of the social status hypothesis (Eisenegger et al., 2011) it is relevant to mention that there was no possibility to increase or restore the social status by aggressing against an opponent, which might be the motivating factor for an actual aggressive response.

Moreover, it is unclear if other emotions may influence the behavior in the non-social frustration task as well. For instance, lower fear in the T compared to the PL group during the neutral block may be an indication of more self-confidence in this task, which might also influence other emotional responses and behavior. While this was not included in the hypotheses, testosterone administration might influence other emotions, such as fear or anxiety, as indicated by observations of behavioral changes in fear-related constructs in previous studies (Aikey et al., 2002; Enter et al., 2016a,b; Wagels et al., 2017a). Also, trait aggression influences behavior in the frustration task. Individuals with high aggression traits are angrier and pull stronger at the joystick when frustrated. Most interestingly, this effect is not enhanced after testosterone administration, but reduced. Testosterone administration may “overrule” the influence of aggressive traits since even individuals with lower aggression traits pull more strongly at the joystick when they are in the frustration block. This is in contrast with previous findings that suggest, that testosterone administration primarily affects individuals who show a tendency to impulsive/ dominant responding (Carré et al., 2017).

The administered dosage of testosterone applied in the current study elevated testosterone levels within the normal range of human males. While we did not test the effects of testosterone administration on the neural system during the frustration task, succeeding tasks suggest that the administration influences neural activity as well (Wagels et al., 2017b, 2019b). Moreover, the effect of acute testosterone increase on the neural system was shown previously *via* a two-step method in which the authors first reduced circulating concentrations of testosterone by applying a gonadotropin-releasing hormone antagonism and then applied 100 mg of a testosterone gel, thereby rapidly increasing testosterone levels within the normal range (Goetz et al., 2014). In their study, the authors show that this acute testosterone increase elevated activity in the subcortical regions including the hypothalamus and corticomedial amygdala when participants saw angry facial expressions.

In the current study, we did not observe an influence of the *MAOA* VNTR on anger related to the frustration task. The *MAOA* polymorphism did not affect the joystick deflections as measured by the size of the peak amplitudes directly, either. However, in *MAOA*-H carriers, a greater deflection was observed in individuals with higher endogenous testosterone levels at baseline. This may seem to be a discrepancy to, the study by Sjöberg et al. (2008), which noticed that increased CSF testosterone levels were associated with lifetime aggression scores in *MAOA*-L carriers, not in *MAOA*-H carriers. Certainly, the current study methodologically differed largely from the study of Sjöberg et al. (2008). First, behavior during a frustration task was measured instead of life-time aggression and testosterone levels were assessed by blood serum samples. Most importantly, although *MAOA* and endogenous testosterone interacted at baseline, testosterone levels were later manipulated in half of the sample. Additionally, the task could have affected endogenous testosterone levels. Thus, the contrasting findings of both studies cannot be directly compared.

Nevertheless, potential biological mechanisms need to be discussed. On the molecular level, evidence that testosterone may directly interact with *MAOA* expression or its substrates is lacking so far in humans. Both genetic variants are thought to affect serotonin regulation (Risch and Nemeroff, 1992; Sabol et al., 1998; Deckert et al., 1999) and serotonin and testosterone seem to have an inverse relationship (Perfalk et al., 2017). Testosterone and serotonin thus might mutually influence aggressive behavior (Birger et al., 2003; Montoya et al., 2012). For a better understanding of the relationship between testosterone and serotonin or testosterone and *MAOA* levels, more studies are needed that investigate such a relationship and investigate how this influences anger and aggression. In addition to suggested molecular relationships, testosterone and serotonin could both influence aggressive behavior *via* different pathways. The serotonergic system may modulate impulsive reactions or behavioral regulation and testosterone may influence emotional reactivity and motivations. An early finding may be interesting in this context: Here, researchers observed that high endogenous testosterone was associated with competitive aggression, but at the same time low CSF concentrations of a serotonin metabolite, reflecting low serotonin turnover, were associated with high rates of aggression such as threats, chases or assaults (Higley et al., 1996). In the current study, high endogenous testosterone levels were associated with increased behavioral responses in the frustration paradigm in *MAOA*-H carriers who are assumed to have a higher serotonin turnover as the transcription rate of *MAOA* is high. This contradictory finding may indicate that the observed effects are not primarily driven by serotonin.

Another *MAOA* metabolite is dopamine, which might be relevant in the context of aggression as well. The *MAOA* polymorphism previously modulated dopamine release in humans while viewing a movie of neutral or violent content (Schlüter et al., 2016). The authors reported an inversed relationship of aggressive behavior and dopamine release as only the *MAOA*-H group showed higher dopamine release and increased aggression after the violent movie while the *MAOA*-L group showed decreased aggressive behavior and no consistent dopamine release. Since in the current study, increased pulling behavior is observed when testosterone levels were higher in *MAOA*-H carriers, this could be an indication for interaction with dopamine release. While both, interactions with serotonin, as well as dopamine and testosterone would be possible, the context could be decisive for observing behavioral effects. Indeed, both in the study of Schlüter et al. (2016), who measured aggression using the PSAP, a well as in the current study, the paradigms had a clear reward (and frustrative non-reward) component which might involve the dopamine system. Frustrative non-reward can also be described as the state that occurs in response to negative prediction errors, which again is known to induce decreases in dopamine neuron firing (Eshel and Leibenluft, 2020). Changes in dopamine firing neurons might be differentially influenced by the *MAOA* variant and might further interact with testosterone levels. The question that remains here would be why the exogenous manipulation could influence emotions but not behavioral responses. If testosterone administration indeed affects the neural system, which can be assumed based on our previous work (e.g., Wagels et al., 2019b), it is unlikely that this would not affect the dopamine system if endogenous testosterone does. Moreover, endogenous testosterone levels would have changed at the time of the experiment. For a better understanding of the relationship between endogenous and exogenous testosterone and its relationship to *MAOA*, it would be advantageous to investigate processes underlying aggressive behavior in a within-subject design, thus being able to compare natural circulating testosterone levels and manipulated testosterone levels within an individual.

Importantly, an assumption of linear or inverse associations might be too simple. Non-linear effects and relationships are possible. Furthermore, other candidate genes are important risk factors of aggression and might certainly contribute to the findings (Beaver et al., 2007; Tielbeek et al., 2012). Nevertheless, it might be important to investigate different types of aggression concerning testosterone and candidate genes for aggression. A better understanding of biological factors underlying the reaction to frustration may also contribute to the understanding of pathological symptoms such as irritability or aggression in psychiatric groups.

## Limitations

The current results have to be interpreted as preliminary results only. Subgroups in the current sample are small to moderate. The power of statistical tests on the interaction may thus have been reduced. Moreover, the current study only includes young healthy young males with no known history of traumatic experiences, which are often discussed in the context of *MAOA* VNTR effects. Previous findings suggest that environmental adversities might influence the effect of the *MAOA* VNTR on aggression (Byrd and Manuck, 2014; Nilsson et al., 2018). Future studies might therefore additionally assess stressful life-events. Also, the *MAOA* gene is X-chromosome linked and thus more complex effects in females can be expected which cannot be investigated in this study, due to a male-only sample. Another limitation of the study is the between-subject design, which does not allow a direct comparison of endogenous testosterone effects with the *MAOA* variant compared to the effects of exogenous testosterone levels with the *MAOA* variant.

## Conclusion

The current study corroborates the influence of testosterone administration on angry emotions in non-social frustration contexts. As a reaction to frustration, testosterone increases anger and overrules the positive effect of trait aggression on joystick pulling behavior increasing impulsive movements also in low aggressive individuals. While not interacting with testosterone administration, the *MAOA* polymorphism modulated the relationship of endogenous testosterone levels at baseline and pulling behavior. *MAOA-H* carriers showed reduced pulling if testosterone levels were low and increased pulling if testosterone levels were high. We thus suggest that in the context of non-social frustration, testosterone administration and *MAOA* operate *via* separate mechanisms, while the *MAOA* polymorphism might influence how endogenous hormones influence behavior.

## Data Availability Statement

The anonymized data supporting the conclusions of this article will be made available by the authors, without undue reservation, to any qualified researcher.

## Ethics Statement

The studies involving human participants were reviewed and approved by the Medical Ethics Committee, Medical Faculty RWTH Aachen. The participants provided their written informed consent to participate in this study.

## Author Contributions

UH, MV and LW designed the study and wrote the protocol. LW managed the literature searches and analyses. PH and LW drafted the manuscript. CM and SJ were responsible for the genotyping analysis. All authors contributed to and approved the final manuscript.

## Conflict of Interest

The authors declare that the research was conducted in the absence of any commercial or financial relationships that could be construed as a potential conflict of interest.

## Footnotes

^1^https://CRAN.R-project.org/
