# Supplementary material for: Single-Dose of Testosterone and the MAOA VNTR Polymorphism Influence Emotional and Behavioral Responses in Men During a Non-social Frustration Task
Source: Front Behav Neurosci. 2020 Jun 25;14:93. doi: 10.3389/fnbeh.2020.00093 (PMC7330109; doi:10.3389/fnbeh.2020.00093)
Supplement: Supplementary file 1 [file Table_1.pdf]

Post hoc exploratory analysis of not anger related emotions

Due to a potential influence of other emotions (happy, sad, fear, surprise) that could be affected in the task and that could be modulated by the hormone administration or the *MAOA* VNTR, we added a repeated measures MANOVA including emotion rating in the neutral and provocation block as well as treatment group and *MAOA* VNTR as influence factors. There was a significant influence of condition showing an increase of sadness, fear and surprise in the frustration block and a decrease of happiness, $F(4,130)= 21.42$, $p< .001$ (table 1). A treatment group by condition interaction, $F(4,130)= 2.79$, $p= .029$, showed significant differences for happiness, $F(4,130)= 5.64$, $p= .019$ and fear, $F(4,130)= 3.99$, $p= .048$. Regarding happiness, there was a stronger decrease of happiness in the testosterone administration group and reduced fear in the neutral block compared to the placebo group (see table 2).

Table 1: Statistics on the main effect of provocation on mood.

Emotion	Provocation condition	M	SE	F	p
happy	n	3.10	0.09	79.14	<.001**
	p	2.40	0.10		
sad	n	1.27	0.06	2.15	.146
	p	1.34	0.06		
fear	n	1.22	0.05	4.09	.045*
	p	1.12	0.04		
surprise	n	2.09	0.10	7.78	.006*
	p	2.36	0.10		

*p<.05, **p<.001. n = neutral condition, p = provocation condition, M = mean, SE = standard error of the mean.
