# Supplementary material for: Single-Dose of Testosterone and the MAOA VNTR Polymorphism Influence Emotional and Behavioral Responses in Men During a Non-social Frustration Task
Source: Front Behav Neurosci. 2020 Jun 25;14:93. doi: 10.3389/fnbeh.2020.00093 (PMC7330109; doi:10.3389/fnbeh.2020.00093)
Supplement: Supplementary file 2 [file Table_2.pdf]

Table 2: Post hoc pairwise comparisons for happiness and fear ratings.

Emotion	level 1	level 2	M	SE	Mean diff level 2	p
happy	PL	n	2.99	0.13	.517	<.001**
		p	2.47	0.14		
	T	n	3.23	0.12	.893	<.001**
		p	2.34	0.13		
fear	PL	n	1.34	0.08	.199	.006*
		p	1.14	0.05		
	T	n	1.09	0.08	.001	.986
		p	1.09	0.05		
happy	n	T	3.23	0.12	.245	.164
		PL	2.99	0.13		
	p	T	2.34	0.13	.131	.495
		PL	2.47	0.14		
fear	n	T	1.09	0.08	.248	.024*
		PL	1.34	0.08		
	p	T	1.09	0.05	.050	.485
		PL	1.14	0.05		

*p<.05, **p<.001. PL = placebo group, T= testosterone group, n = neutral condition, p = provocation condition, M = mean, SE = standard error of the mean. Significance is indicated for the pairwise comparison across level 1.
