# Supplementary material for: Single-Dose of Testosterone and the MAOA VNTR Polymorphism Influence Emotional and Behavioral Responses in Men During a Non-social Frustration Task
Source: Front Behav Neurosci. 2020 Jun 25;14:93. doi: 10.3389/fnbeh.2020.00093 (PMC7330109; doi:10.3389/fnbeh.2020.00093)
Supplement: Supplementary file 3 [file Image_1.pdf]

Genotyping

DNA from buccal mucosa cell samples was analyzed in a collaborate laboratory (Molecular Psychology, Ulm, Germany).

Genomic DNA was extracted from buccal cells using the MagNAPure 96 System and a commercial extraction kit (MagNa Pure 96 DNA Kit; Roche Diagnostics, Mannheim, Germany). Amplification of the MAOA VNTR sequence was performed via polymerase chain reaction PCR (30s at 95 °C, 30s at 58,4 °C, 60s at 72 °C for 30 cycles with an initial denaturation step of 5 min at 95 °C and a final elongation step of 5min at 72 °C). PCR products were separated by electrophoresis on a 2% high resolution agarose gel and visualized by ethidium bromide staining. All gel electrophoresis runs were performed with controls of different repeat variants. The repeat numbers of the controls and ambiguous samples were checked by matters of fragment length analysis using FAM-labeled forward primers. Opening primer sequences for the 30-bp VNTR in the promotor region of the MAOA region frame were: forward, 5'ACAGCCTGACCGTGGAGAAG-3'; and reverse, 5'-GAACGTG ACGCTCCATTCGGA-3' (1). Different to the findings of Sabol and colleagues (1) we did not find a 3.5 repeat variant. Instead of the 3.5 (3 repeats + 15bp) we detected a 3a variant (3 repeats + 18 bp) variant consistent with others (2). The term "3a" is used by Deckert and colleagues (2). Alleles with repeat sizes 3R (314 bp), 3a repeats (332 bp) and 4 repeats (344 bp) were included, with the most common being the 3R (314 bp) and 4R (344 bp) alleles.

Joystick amplitude

For visual purposes the peak amplitudes of the joystick are depicted for each trial (figure 1). The figures demonstrates that individuals in the neutral trial pulled less strongly over time, while they pulled more strongly in the frustration block.

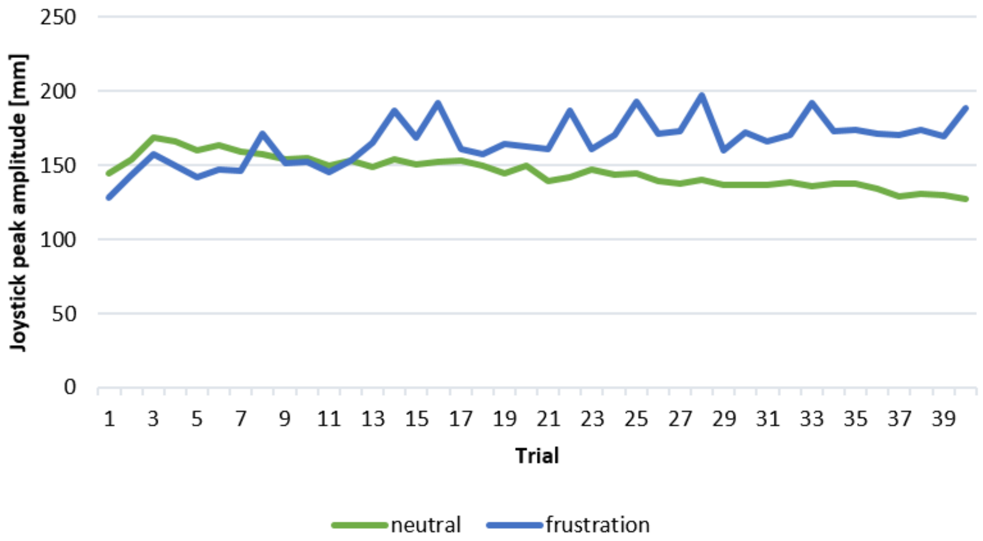

Figure 1: Mean peak amplitude for neutral and frustration block.
